# Use of *Lactobacillus crispatus* to produce a probiotic cheese as potential gender food for preventing gynaecological infections

**DOI:** 10.1371/journal.pone.0208906

**Published:** 2019-01-09

**Authors:** Francesca Patrignani, Lorenzo Siroli, Carola Parolin, Diana I. Serrazanetti, Beatrice Vitali, Rosalba Lanciotti

**Affiliations:** 1 Department of Agricultural and Food Sciences, Alma Mater Studiorum—University of Bologna, Cesena, Italy; 2 Department of Pharmacy and Biotechnology, Alma Mater Studiorum—University of Bologna, Bologna, Italy; Maharshi Dayanand University, INDIA

## Abstract

This research is aimed to evaluate the suitability of Squacquerone cheese to support the viability of *Lactobacillus crispatus* BC4, a vaginal strain endowed with a strong antimicrobial activity against urogenital pathogens and foodborne microorganisms, in order to recommend a gender food for woman wellbeing. The viability of *L*. *crispatus* BC4, used as adjunct culture, was evaluated during the refrigerated storage of Squacquerone cheese, as well as when the cheese was subjected to simulated stomach-duodenum passage tested by the patented Simulator of the Human Intestinal Microbial Ecosystem (SHIME). Moreover, the effects of *L*. *crispatus* BC4 addition were evaluated on product hydrolytic patterns, in terms of proteolysis, lipolysis and volatile molecule profiles. The data showed that *L*. *crispatus* BC4 maintained high viability, also in presence of physiological stress conditions, until the end of the refrigerated storage. Moreover, the inclusion of *L*. *crispatus* BC4 gave rise to cheese product with higher score of overall acceptability when compared to control cheese. In addition, the survival of *L*. *crispatus* BC4, carried in test cheese, in gastro intestinal conditions was confirmed by SHIME. The results showed that the vaginal *Lactobacillus* strain was more affected by the low pH of the stomach, simulated by the SHIME reactor, rather than to bile salts and pancreatic juices. Although only in vivo trials will be able to confirm the functionality of the cheese in the vaginal environment, these data represent a first step towards the employment of the Squacquerone cheese as probiotic food able to promote the woman’s health by preventing gynaecological infections.

## Introduction

The demand of the consumers in food production has considerably changed throughout the years. In this time, foods can be perceived not only to satisfy hunger but also to directly contribute to consumer’s health providing necessary nutrients, and preventing nutrition-related diseases and improving the general human well-being [1]. In this framework, one of the fastest growing areas in the global food industry is represented by functional foods [2] considered as dietary strategy to reduce the incidence of illness for humans. The European Commission's Concerted Action on Functional Food Science in Europe, coordinated by the International Life Science Institute, described functional foods as “*products having beneficial effects on one or more functions of the human organism thus either improving the general and physical conditions or/and decreasing the risk of the evolution of diseases*. *The amount and form of the functional food should be for dietary purposes*. *Therefore*, *it could not be in pill or capsule but as food form*”. Many functional foods are characterized by health promoting features because they are vehicle of probiotic microorganisms, which are recognized as able to provide beneficial effects to the host, positively affecting the intestinal microflora balance, reducing the growth of pathogens, producing and increasing the bioavailability of nutrients, favouring good digestion, decreasing the effect of allergens, stimulating the human immune system, lowering cholesterol, alleviating lactose intolerance, and increasing infection resistance [3–6]. The rationale for the use of probiotics, independently on the vehicle used for their intake, relies mainly on their regulatory role in modulating the gastrointestinal and genitourinary microbiota [7]. Substantial amount of emerging research is indicating that the microbiota has a significant impact on human health [8–15]. According to some updated papers, humans can be regarded as “holobionts” or communities made of the host and their symbiotic microbes, rather than individuals, since the microbiome has a fundamental role in human physiology. Indeed, the interaction between host genome and microbiome increases the genetic variation and phenotypic plasticity, thus enabling the holobiont to improve its general health [16–18]. Current exciting papers are beginning to underline how foods, with their components and microbial communities and probiotics, modulate the human symbiotic microbes [19,20].

Indeed, due to many physiological gender differences (including the physiological responses to many pathologies), diet and probiotic foods are expected to differently influence the male and female health though the modulation of their symbiotic microbes [18]. Until the last decade, research on women has been ignored and the results coming from men were directly turned to women in several medical fields [21]. However, some studies and randomized controlled trials devoted to the female gender, demonstrated the capability of probiotics, used as oral or vaginal supplements, to recover from glycemic disorder in healthy pregnant women, infectious mastitis, bacterial and *Candida* genitourinary infections [7, 22–24]. Moreover, some literature data showed the suitability of functional foods to prevent heart disease in post-menopausal ages, improve the woman skin health, and positively affect the gut and vagina microbiota of HIV positive women [25–28].

Reproductive-age women are often subjected to abnormalities in vaginal microbiota, leading to gynaecological infections, such as vulvovaginal candidiasis, bacterial vaginosis, aerobic vaginitis and sexually transmitted diseases [29]. These disturbances interfere with female reproductive health, promoting abortion, preterm delivery, premature rupture of membranes and chorioamnionitis. The literature data have shown that treatments based on conventional approaches were not successful in the handling of these disorders, and that the use of probiotics can be effective in restoring the normal vaginal microbiota dominated by lactobacilli [30].

Recent studies highlighted the anti-microbial activities of *Lactobacillus* strains isolated from vagina of healthy women and their potential appliance for the production of probiotic formulations [31, 32]. In particular, some *Lactobacillus crispatus*, *Lactobacillus gasseri* and *Lactobacillus vaginalis* strains were found to be active towards vaginal *Candida* isolates, common urogenital pathogens and the sexually transmitted agents *Chlamydia trachomatis*, *Neisseria gonorrhoeae* and HIV [33–36]. Those strains were also characterised for some functional and technological properties [36] in order to evaluate their potential inclusion in dairy products. Several strains of *L*. *crispatus* showed a significant antagonistic activity against spoilage and pathogenic microorganisms of food interest, their fermentation kinetics in milk and their ability to survive at 4°C suggested their potential use as adjunct cultures for the production of female gender foods. In this view, the production of a functional gender food based on *L*. *crispatus* represents a new strategy to vehicle a health-promoting strain to the vaginal environment. This idea is supported by the already demonstrated ability of probiotic strains to pass from intestine to vagina [37–41]. In this scenario, the main aim of this research was the evaluation of the suitability of a soft cheese like Squaquerone to serve as a carrier of *L*. *crispatus* BC4, a selected vaginal strain gifted of interesting antimicrobial and technological properties [31,36]. The effects of the inclusion of this *Lactobacillus* strain on the cheese safety, shelf-life, proteolytic and lipolytic patterns, volatile molecule and sensory profiles were evaluated, and compared to the traditional product. In addition, the viability of this strain during refrigerated storage, simulated gastro-duodenum passage and in the patented simulator of the Human Intestinal Microbial Ecosystem (SHIME) was evaluated since the ability to survive through the gastrointestinal tract is notoriously fundamental for the strain functionalities in the host. About this, SHIME system has been used as for the accurate simulation in vitro on the ability of probiotic bacteria to merge into the intestinal environment [42]

## Materials and methods

### *L*. *crispatus* and cheese starter culture conditions

*L*. *crispatus* BC4, a vaginal isolate able to contrast *Candida* spp. and other pathogens of the urogenital tract [31,33,36], was used in this study for the production of a test cheese. Overnight cultures (37°C for 16 h) of *L*. *crispatus* were obtained in de Man-Rogosa-Sharpe (MRS) broth (Oxoid, Basingstoke, UK) in anaerobic condition by using Oxoid gas generating kit. Cells were harvested by centrifugation at 8,000 × g for 20 min at 4°C. The resultant pellet was washed twice with saline solution (0.9% NaCl in distilled water) and resuspended in commercial milk for the inoculums in industrial environment. A commercial freeze-dried culture of *Streptococcus thermophilus* St 0.20 (Sacco S.R.L., Como, Italy) was used as starter for Squacquerone cheese production. The culture was inoculated in milk in the optimal conditions and in the proportions indicated by the producer.

### Squacquerone cheesemaking

The production of the Squacquerone cheeses (test cheese supplemented with *L*. *crispatus* BC4 and control cheese) was carried out in a pilot-scale plant of a local dairy farm (Mambelli, Bertinoro, Italy) For each cheese type, 100 liters of milk were heated and maintained at 42°C and inoculated with the commercial starter culture of *S*. *thermophilus* (final concentration: 6 log CFU/mL). To obtain test Squacquerone cheese, *L*. *crispatus* BC4 was added (final concentration: 6.8 log CFU/mL). After 40 minutes, the two batches were added of NaCl (0.7%) and 37 mL of rennet (12000 U, 80% chymosin and 20% pepsin, Bellucci Modena, Italy). Twenty min after coagulation, the curd was cut and transferred into traditional moulds. The cheeses were let to rest until the reaching of pH 5.15 and stored at 4°C. After 1 day, the cheeses were packed in modified atmosphere and stored at 4°C for 18 days.

### Microbiological analyses, pH and water activity

The test cheese supplemented with *L*. *crispatus* BC4 and the control cheese (without *L*. *crispatus* BC4) were subjected to microbiological, pH and water activity analyses. For the microbiological analyses, 20 grams of cheese was placed into 180 mL sodium citrate sterile solution (20 g/L) and homogenised in a stomacher (Lab-blender 80, Pbi International, Milan, Italy) for 3 min. Decimal dilutions were performed in Ringer’s solution (NaCl 0.9%), and 0.1 mL of appropriate dilutions was spread onto different agar media. *S*. *thermophilus* was counted on M17 agar (Oxoid, Basingstoke, Hampshire, UK) (42°C, 48 h), *Lactobacillus* spp. were detected on MRS agar (37°C, 48 h, anaerobic conditions) and the presence of *L*. *crispatus* was assured also by checking the bacterial colony morphology. To confirm its presence, genomic DNA was extracted from each *L*. *crispatus* presumptive colony using the InstaGene Matrix kit (Bio-Rad Laboratories, Milano,Italy) and by sequencing the 16S rRNA region according to the protocol described by De Angelis et al. [43]. *Enterobacteriaceae* and yeasts were also counted on VRBGA (Oxoid, Basingstoke, Hampshire, UK) (37°C, 24 h) and YPD (Oxoid, Basingstoke, Hampshire, UK) (25°C, 48 h) plates, respectively. The presence of pathogenic species such as *L*. *monocytogenes*, *S*. *enteritidis* and *E*. *coli* was checked in both the cheeses during their refrigerated storage, according to the ISO methods 11290, 6579, and 16649, respectively.

pH and water activity were measured by diluting 10 g of cheese with 10 mL of distilled water. pH was registered by using a pH-meter Hanna Instruments 8519 (Incofar, Modena, Italy). Water activity (Aw) was measured by using an Aqualab Series 4TE (Decagon Device, Inc. Pullman,WA, USA).

### Proteolysis, lipolysis, volatile profiles

The test cheese supplemented with *L*. *crispatus* BC4 and the control cheese (without *L*. *crispatus* BC4) were subjected to proteolysis, lipolysis and volatile profile. The proteolysis of the two cheese types was monitored by SDS-PAGE. The soluble proteins at pH 4.6 were extracted from the supernatants of the two cheese types following the method proposed by Kuchroo and Fox [44] while the running conditions adopted are in accordance to Tofalo et al. [45]. The extraction of cheese lipids and the determination of free fatty acid (FFA) concentrations were performed as described by Vannini et al. [46]. The main volatile compounds were monitored by using a GC/MS/SPME technique according to the method proposed by Burns et al. [47]. The compounds were identified by use of available mass spectra databases (NIST version 2005).

### Textural profile analyses

The test cheese supplemented with *L*. *crispatus* BC4 and the control cheese (without *L*. *crispatus* BC4) were subjected to textural analysis over the refrigerated storage. Texture analyses were performed after 1, 4, 6, 8 and 13 days of refrigerated storage using a Texture Analyser TA DHI (Stable MicroSystem, Godalming, UK) and according to the method proposed by Patrignani et al. [2].

### Organoleptic evaluation

The test cheese supplemented with *L*. *crispatus* BC4 and the control cheese (without *L*. *crispatus* BC4) were subjected to sensory evaluation over the refrigerated storage. The sensory evaluation was performed throughout a panel test after 4 and 13 days of storage at 4°C. Twenty-five trained panellists were enrolled within the employees of Mambelli (Bertinoro, Italy) local dairy farm (5 panellists) and of the Department of Agricultural and Food Sciences, University of Bologna, Campus Food Science, Cesena, Italy (20 panellists). The Campus of Food Science, where the test was organized, is provided by sensory-laboratories certified according to regional requirements. The panellists were verbally informed on the cheese product manufacture and the strains used in the research by the committee organizing the test. The committee was formed by Dr. Patrignani, Dr. Siroli and Prof. Lanciotti. The panellists, after oral consent to perform the test, tasted 20 g of each sample served at 15°C under controlled conditions according to Standard 8589 (ISO, 1988), as suggested by Gallardo-Escamilla et al. [48]. The assessors evaluated cheese colour, flavour, creaminess, off-flavours, bitter and overall acceptance attributing a score ranging from 0 (low or poor) to 5 (high or very excellent).

### Evaluation of the fate of Lactobacilli population (mainly represented by *L*. *crispatus* BC4) by Simulated stomach duodenum passage and the Simulator of the human intestinal microbial ecosystem (SHIME)

The fate of *L*. *crispatu*s BC4 was performed by the simulation of the passage through stomach and duodenum, developed according to the method proposed by Vinderola et al.[49]. Briefly, 25 g of tested cheese was mixed with the same volume of a ‘saliva-gastric’ solution. Saliva-gastric solution contained CaCl_2_ (0.22 g⁄L), NaCl (16.2 g⁄L), KCl (2.2 g⁄L), NaHCO_3_ (1.2 g⁄L) and 0.3% (w⁄v) porcine pepsin. Porcine pepsins from two different and undisclosed manufacturers were used on a g⁄mL basis because no information about the enzymatic units was available on the label of the products. A 1 mL sample was removed for cell counts immediately after admixture and pH was quickly brought to 3.00, 2.70 or 2.50, with 5 M and 0.1 M HCl. Samples were brought to 37°C in a water bath. Aliquots (1 mL) were taken at the time fixed by the method and serial dilutions were plated for cell viability assessment.

The destiny of the vaginal strain *L*. *crispatus* BC4 during passage in the human upper gastrointestinal tract when delivered within a cheese matrix was evaluated also in an adapted SHIME system [50]. This system, composed of a reactor used in a sequential setup to simulate over time first the stomach and then the small intestinal environment, has been used as for the accurate simulation in vitro on the ability of probiotic bacteria to merge into the intestinal environment [42].

Appropriate retention time and pH were chosen in order to resemble *in vivo* conditions in the different parts of the gastrointestinal tract in fed state. Control and test cheeses were incubated as reported below to simulate gastric and upper intestinal phases, both working in fed state. 2.5 g of each cheese were used in the simulation, all experiments were conducted in triplicate to account for biological variability. Along the simulation, the following samples were collected: time 0, end of stomach, small intestine after 1, 2 and 3h. Viability of the *L*. *crispatus* strain was evaluated by means of conventional culturing techniques using selective medium (MRS). To confirm its presence, the procedure previously described was used [43]. The SHIME consists of a succession of reactors simulating the different parts of the human gastrointestinal tract. In this research, the SHIME reactor simulates over time, firstly the stomach and then the small intestine. The applied conditions used in the simulator are summarized below:

Gastric phase (fed state): the cheese matrix was incubated at 37°C for 2 h at the SHIME patent conditions [50], while mixing via stirring, with sigmoidal decrease of the pH profile. Pepsin was supplied with the activity being standardized by measuring absorbance increase at 280nm of TCA-soluble products upon digestion of hemoglobin (reference protein). Phosphatidylcholine was added and followed by the addition of SHIME complex nutritional medium. The salt (NaCl and KCl) levels were implemented according to Mackie et al. [51].

Small intestinal phase (fed state): The small intestine phase was characterized by an incubation of the cheese matrix, after gastric phase, at 37°C for 3 h, while mixing via stirring. In this step, pH increased to 7.4. Pancreatic enzymes, in a specific and defined ratio, were used according to the patent. 10 mM bovine bile extract was supplemented.

### Statistical analysis

The data are expressed as the mean of three repetitions and two independent experiments. Microbiological, chemico-physical and texture data were analyzed using Statistica software (version 8.0; StatSoft, Tulsa, Oklahoma, USA). Means were compared using ANOVA followed by LSD test at p < 0.05 level to mark differences between samples with the same storage times. The volatile molecule profiles were analyzed using ANOVA followed by a principal component analysis (PCA) performed by Statistica software.

## Results

### Microbiological analyses

**Fig 1:**
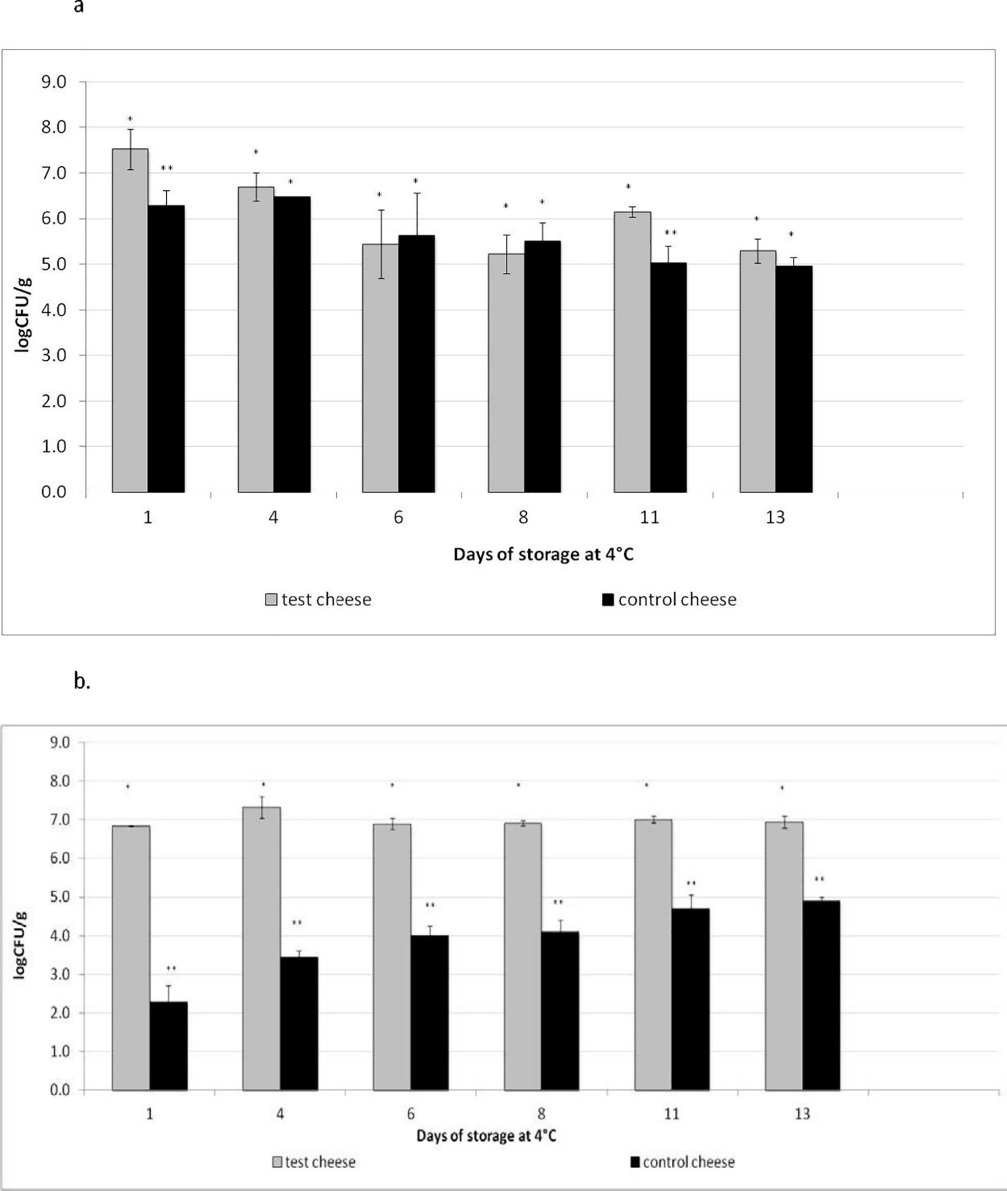
Evolution of *Streptococcus thermophilus* (a) and *Lactobacillus* population (b) concentrations in test and control Squacquerone cheese during the refrigerated storage (1, 4, 6, 8, 11, 13 days). Cell loads are expressed as mean Log CFU/g cheese ± standard deviation. Control and test cheese cell loads were statistically compared at each sampling time. Comparing the same day of sampling, cell loads values of test and control cheeses having different symbols are significant different (p <0.05).

Test Squacquerone cheeses were produced in the pilot-scale plant of a local Italian cheesemaker using *L*. *crispatus* BC4 strain as adjunct culture with the starter one of *S*. *thermophilus*. The test cheeses were compared to the control ones, produced in the same conditions with the starter culture alone. The cell loads of the starter (*S*. *thermophilus*) and of *Lactobacillus* population were monitored during the refrigerated storage (13 days) and reported in Fig 1, respectively. The results highlighted the constant presence of *S*. *thermophilus* over the whole storage period, independently on the presence of the adjunct culture. Notably, the inclusion of *L*. *crispatus* BC4 in the cheese positively affected the starter culture viability over the storage. In fact, in the test cheese, the initial count (1d) of *S*. *thermophilus* was 7.4 log CFU/g and decreased to 5.9 log CFU/g at the end of the storage (13d), while in the control one, *S*. *thermophilus* had an initial cell load of 6.1 log CFU/mL and it reduced to 5.0 log CFU/g (Fig 1A). Fig 1B reports the cell loads of *Lactobacillus* population over the storage period. The levels of lactobacilli in the control cheese increased from 2.1 log CFU/g (1d) to 5.0 log CFU/g (13d). By contrast, lactobacilli cell load was quite constant in the test cheese and always over 7.0 log CFU/g. According to the morphological and molecular analysis, the most abundant *Lactobacillus* population was represented by *L*. *crispatus* BC4, that was inoculated at a level of 6.8 log CFU/g. On the contrary, *L*. *crispatus* was not found among the *Lactobacillus* population detected in the control cheese. No contamination by yeasts, *Enterobacteriaceae* and pathogenic species was found.

### Physico-chemical analyses

The pH and water activity values of Squacquerone cheeses, in relation to storage time and *L*. *crispatus* BC4 presence, are reported in Table 1. During the storage, the pH decreased both in test and control cheese, even if, at the end of the storage, the pH of the cheese containing *L*. *crispatus* BC4 was lower than the control. Regarding water activity, the two cheese types presented significant different values at the beginning of the shelf-life while after 13 d of storage no significant difference was recorded between the two cheese types.

**Table 1:** Evolution of pH and water activity in test and control cheese over refrigerated storage.

	Storage (d)	pH	aw
Test cheese	1	5.35 ± 0.01^a^	0.987±0.001^a^
	4	5.45 ± 0.02 ^a^	0.991±0.001^a^
	6	5.38 ± 0.01^a^	0.992±0.001^a^
	8	5.35 ± 0.01^a^	0.993±0.001^a^
	13	5.10 ± 0.01^a^	0.991±0.001^a^
Control cheese	1	5.29 ± 0.02^b^	0.972±0.001^b^
	4	5.27 ±0.01 ^b^	0.991±0.002^a^
	6	5.28 ± 0.03^b^	0.998±0.001^b^
	8	5.28 ±0.02^b^	0.997±0.001^b^
	13	5.22 ± 0.03^b^	0.992±0.001^a^

At each sampling time, pH and water activity values of test and control cheeses were statistically compared. Comparing the same day of sampling, physic-chemical values of control and test cheeses having different capital letters (^a^ or ^b^) were significant different (p<0.05).

### Cheese ripening profiles

**Fig 2:**
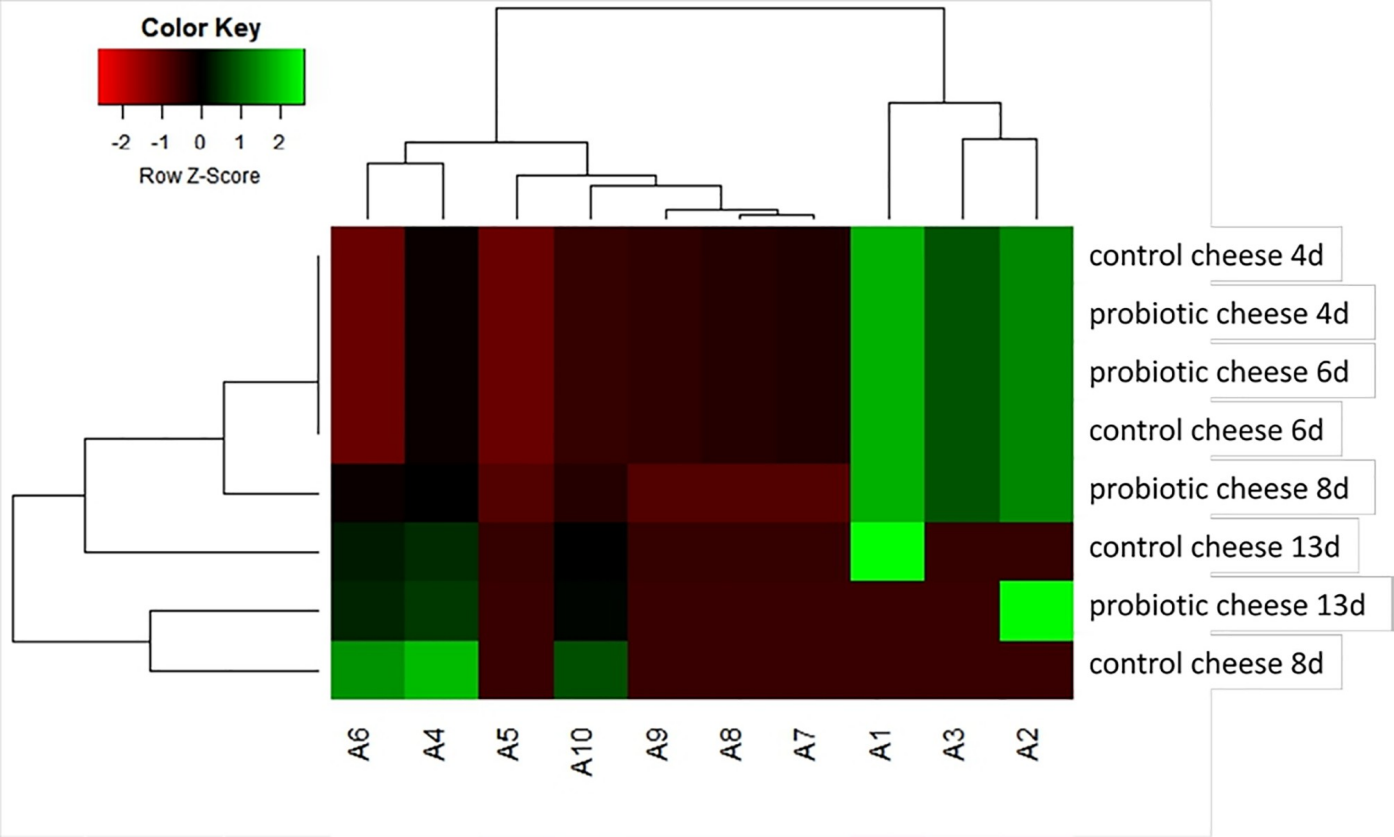
Heat-map relative to the proteolitic patterns detected in control and test Squacquerone cheeses in relation to the refrigerated storage time (4, 6, 8, 13 days). Protein molecular weight: A1: 78.39 KDa; A2: 66.00 KDa; A3: 51.56 KDa; A4: 25.22 KDa; A5: 23.92 KDa; A6: 21.07 KDa; A7: 19.57 KDa; A8: 17.81 KDa; A9: 15.14 KDa; A10: 14.18 KDa.

The proteolytic profiles of the two types of Squacquerone cheese, containing or not *L*. *crispatus* BC4, are represented in the heat map reported in Fig 2. The dendrogram of the cheese proteins soluble at pH 4.6 showed similar weight molecular bands for the two cheese types up to 6 days of storage. As the storage time increased, significant differences between the test and control cheeses were detected. In fact, after 13 days of refrigerated storage, the cheese containing *L*. *crispatus* BC4 showed a proteolytic profile very similar to that of the control cheese after 8 days of storage, with the exception of the band corresponding to 66 KDa. This protein band, corresponding to the molecular weight of bovine serum albumin, characterized the test cheese while it was absent in the control cheese, suggesting that the activity of the adjunct culture affected the matrix protein network and the whey separation and, consequently, the retention of whey proteins. This hypothesis is also corroborated by the rheological data showing that cheese containing *L*. *crispatus* BC4 was characterized by a significantly higher creaminess, with respect to the control cheese, also immediately after cheesemaking. Also the faster decrease of the pH during storage in test cheese probably contributed to modify the water binding capacity of the proteins and their interaction with the other macromolecules of the system. In fact, the pH has a key role in the interaction between proteins and other macromolecules [52]. By contrast, the low molecular weight bands differed between the two cheese typologies only for their abundance (Row-Z-score). More specifically, peptides having molecular weights ranging between 21.07 and 14.18 KDa were more abundant in the control cheese.

The cheese FFA contents recorded between 4 and 13 days of storage at 4°C, are shown in Table 2. After 4 days of storage, the control cheese presented FFA profiles characterized by C12:0, C14:0, C16:0, and C18:1, while the test cheese was characterized by C10:0, C14:0, C16:0, C18:0 and C18:1.Over the storage, in both cheese typologies the release of FFA increased, even though with different trends in relation to the presence of *L*. *crispatus*. The C18:0 was detected in the control cheese after 6 days of storage. Branched anteiso 17 carbon atoms, as well as long-chain fatty acids such as C18:2 and C20:0 appeared in test cheese type between 6 and 13 days of storage. Both in the control and in the test cheese, also 15 carbon atom FFAs and C18:2 appeared after 6 days of storage. However, the presence of *L*. *crispatus* BC4 fastened the release of FFAs, whose concentrations peaked at 8 days of storage, and then decreased probably due to their further transformation in aroma compounds. By contrast, in control cheese the accumulation of the reported FFAs continued over time and peaked at the end of storage.

**Table 2:** Free fatty acids (ppm ± standard deviation) detected in the two types of Squacquerone cheese in relation to the storage time.

	Test cheese				Control cheese			
	4d	6d	8d	13d	4d	6d	8d	13d
(C10:0) Decanoic acid, methyl ester	2.2±0.1	2.2±0.2	-	3.6±0.3	-	-	-	3.0±0.2
(C12:0) Methyl dodecanoate	-*	3.1±0.3	2.5±0.1	5.4±0.2	2.7±0.2	2.2±0.1	2.8±0.2	3.3±0.1
(C14:0) Methyl tetradecanoate	4.4±0.5	10.2±0.5	11.7±0.4	20.0±1.1	7.3±0.2	8.2±0.2	9.8±0.4	10.8±0.1
(C15:0) Methyl pentadecanoate	-	-	2.7±0.2	3.8±0.3	-	2.1±0.1	2.6±0.1	2.6±0.2
(C16:1) Methyl cis-9-hexadecenoate	-	-	-	2.6±0.2	-	-	-	-
(C16:0) Methyl hexadecanoate	44.0±1.5	89.1±2.1	122.7±2.9	224.4±3.0	68.4±0.9	96.6±2.3	109.6±2.2	127.4±11
(a-17:0) Methyl 14-methylhexadecanoate	-	-	2.2±0.1	-	-	-	-	-
(C17:0) Methyl heptadecanoate	-	2.1±0.1	2.3±0.1	3.5±0.2	-	-	2.1±0.1	2.3±0.1
(C18:2) Methyl cis-9,12-octadecadienoate	-	-	2.1±0.1	2.9±0.1	-	-	2.1±0.1	3.6±0.2
(C18:1) Methyl cis-9-octadecenoate	5.2±1.0	7.6±0.3	19.8±1.3	18.9±0.2	6.0±0.3	6.2±0.1	10.7±1.0	12.2±1.2
(C18:1) Methyl trans-9-octadecenoate	-	-	2.5±0.1	-	-	-	-	-
(C18:0) Methyl octadecanoate	44.6±1.8	89.7±2.4	134.3±3.4	103.4±1.9	-	80.2±2.6	98.3±1.4	134.2±3.1
(C20:0) Methyl eicosanoate	-	-	2.4±0.3	2.2±0.1	-	-	-	2.1±0.1
**TOTAL FFA**	**100.4**±4.6^a^	**204.0**±5.9^a^	**305.2**±8.9^a^	**290.7**±7.3^a^	**84.4**±1.8^b^	**195.5**±5.3^a^	**238.0**±7.2^b^	**301.5**±6.2^a^

*under the detection limit

At each sampling time, total FFA values of test and control cheeses were statistically compared. Comparing the same day of sampling, total FFA values of control and test cheeses having different capital letters (^a^ or ^b^) were significant different (p<0.05).

**Fig 3:**
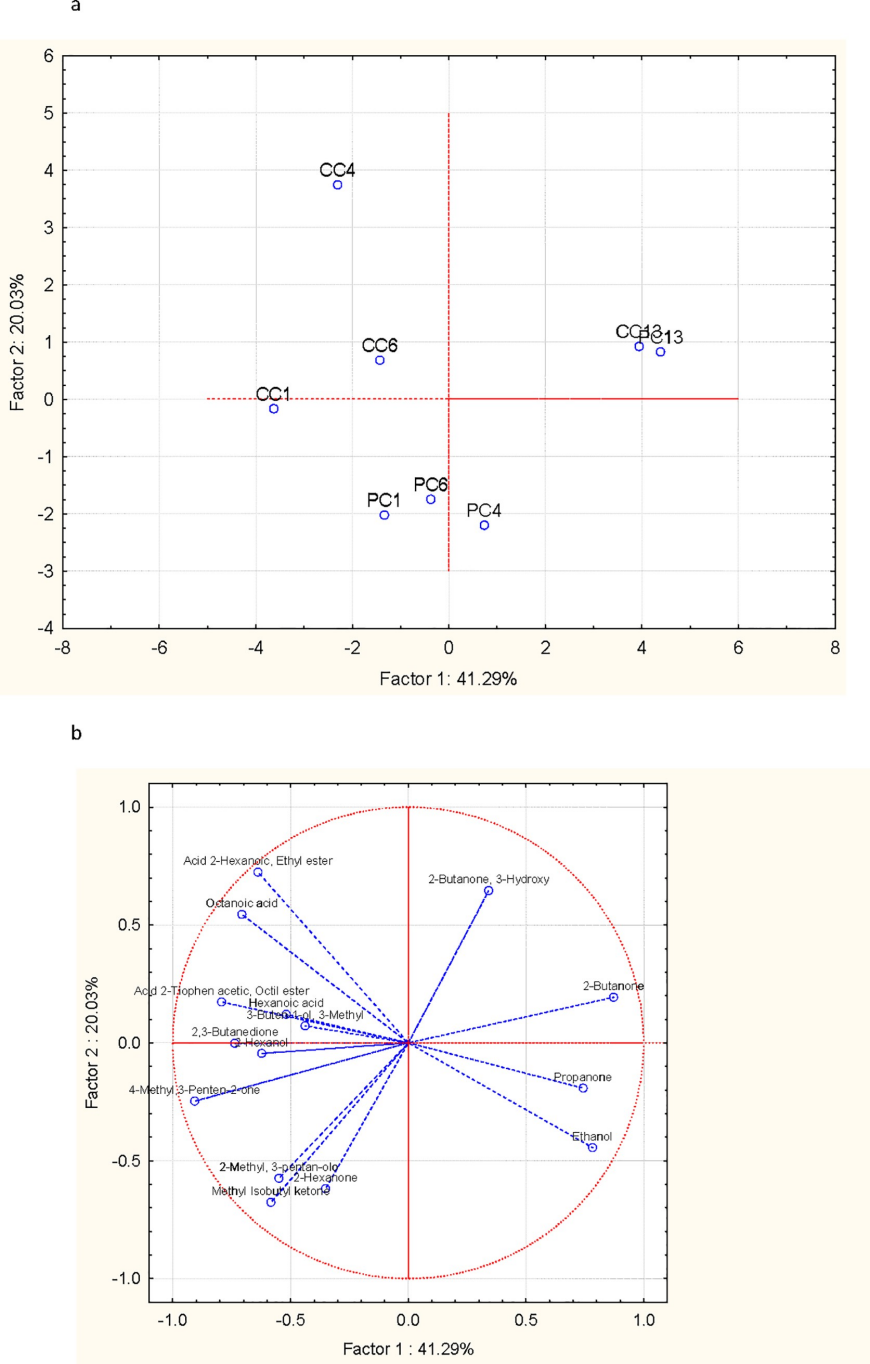
Plot of cases (a) and variables (b) obtained by PCA elaboration of the total volatile molecules characterising control and test Squacquerone cheeses in relation to the refrigerated storage time (1, 4, 6, 13 days). CC1: control cheese after 1 day of storage; CC4: control cheese after 4 days of storage; CC6: control cheese after 6 days of storage; CC13: control cheese after 13 days of storage; PC1: Test cheese after 1 day of storage; PC4: Test cheese after 4 days of storage; PC6: Test cheese after 6 days of storage; PC13: Test cheese after 13 days of storage.

The volatile molecule profiles of the two types of Squacquerone cheese were monitored over the refrigerated storage using the GC/MS/SPME technique. The detected molecules belonged to different classes of compounds such as alcohols, esters, acids and ketones. To evaluate the consequences of the inclusion of *L*. *crispatus* BC4 over time, the detected volatile compounds were analysed using a principal component analysis (PCA). In Fig 3, the projection of samples and detected molecules is reported. The PCA was able to explain over 60% of the total variance among the samples over time. All the test cheese samples, independently on the time of storage, clustered together, with the exception of the test cheese after 13 days of storage that was very similar to the control one at the same time of storage. At 1, 4 and 6 days of storage, the test cheese was principally characterized by ketones and alcohols, while the control cheese was characterized by ketones, short fatty acids and esters. At the end of the shelf-life, 2-butanone, propanone and, at minor amount, ethanol were found in both types of cheese.

### Panel test

**Fig 4:**
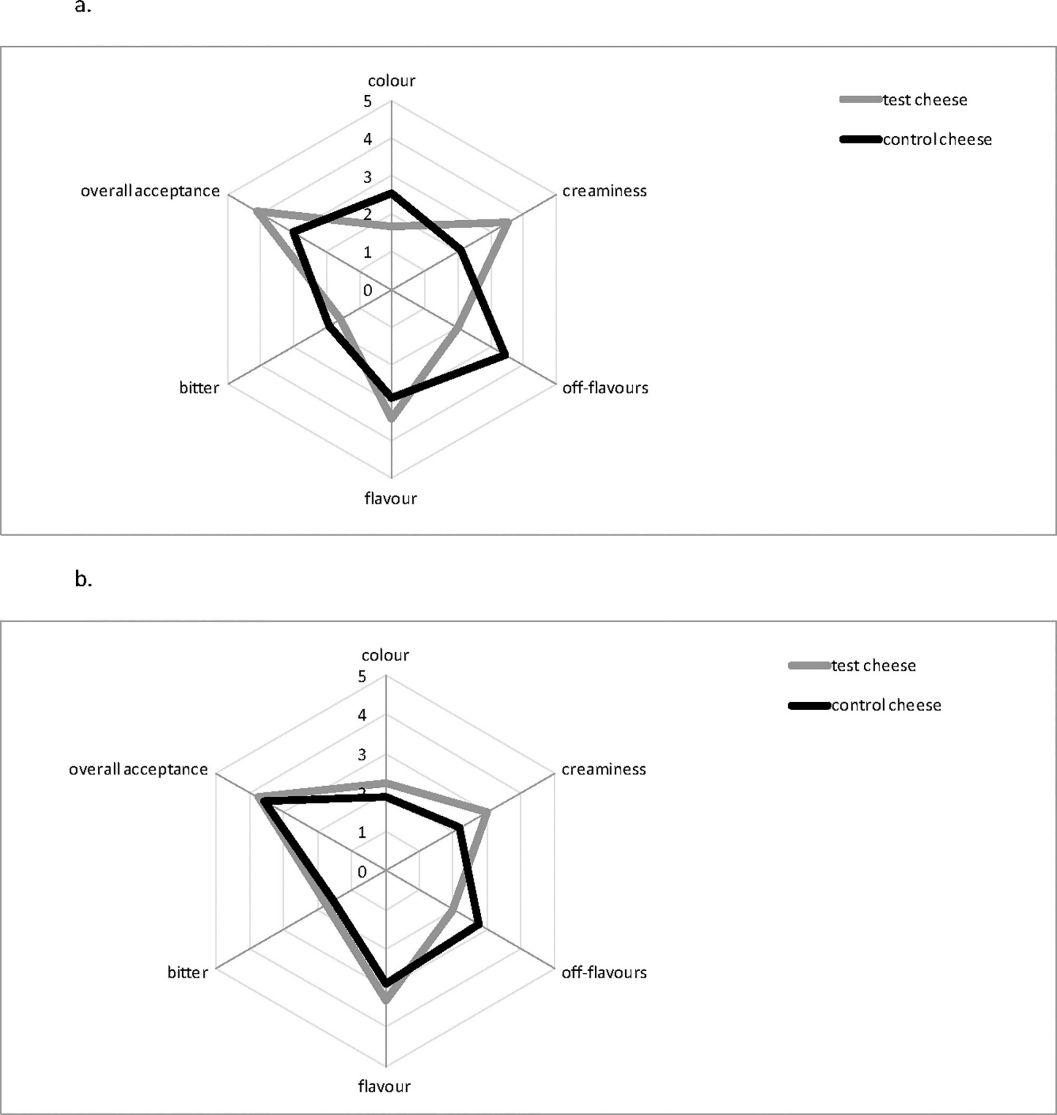
Spider web chart of the sensory data of control and test Squacquerone cheeses after 4 (a) and 13 (b) days of refrigerated storage. The sensory evaluation of the samples, assessed by a trained assessment panel, considered cheese colour, flavour, creaminess, off-flavours, bitter and overall acceptance attributing a score ranging from 0 (poor) to 5 (very excellent).

The sensory analysis was performed on the two types of cheese after 4 and 13 days of storage and showed that test cheese was preferred compared to the control (Fig 4). In fact, after 4 days, test cheese received a significant higher score compared to the control cheese for the overall acceptance. This positive evaluation was principally affected by sensory features such as creaminess, flavour, and lacking of off-flavours, balancing the low scores received for the colour attribute. After 13 days of storage, the sensory differences perceived by the panellists were significantly reduced, although the test cheese still remained more appreciated than the control for all the parameters considered.

### Textural profile

**Fig 5:**
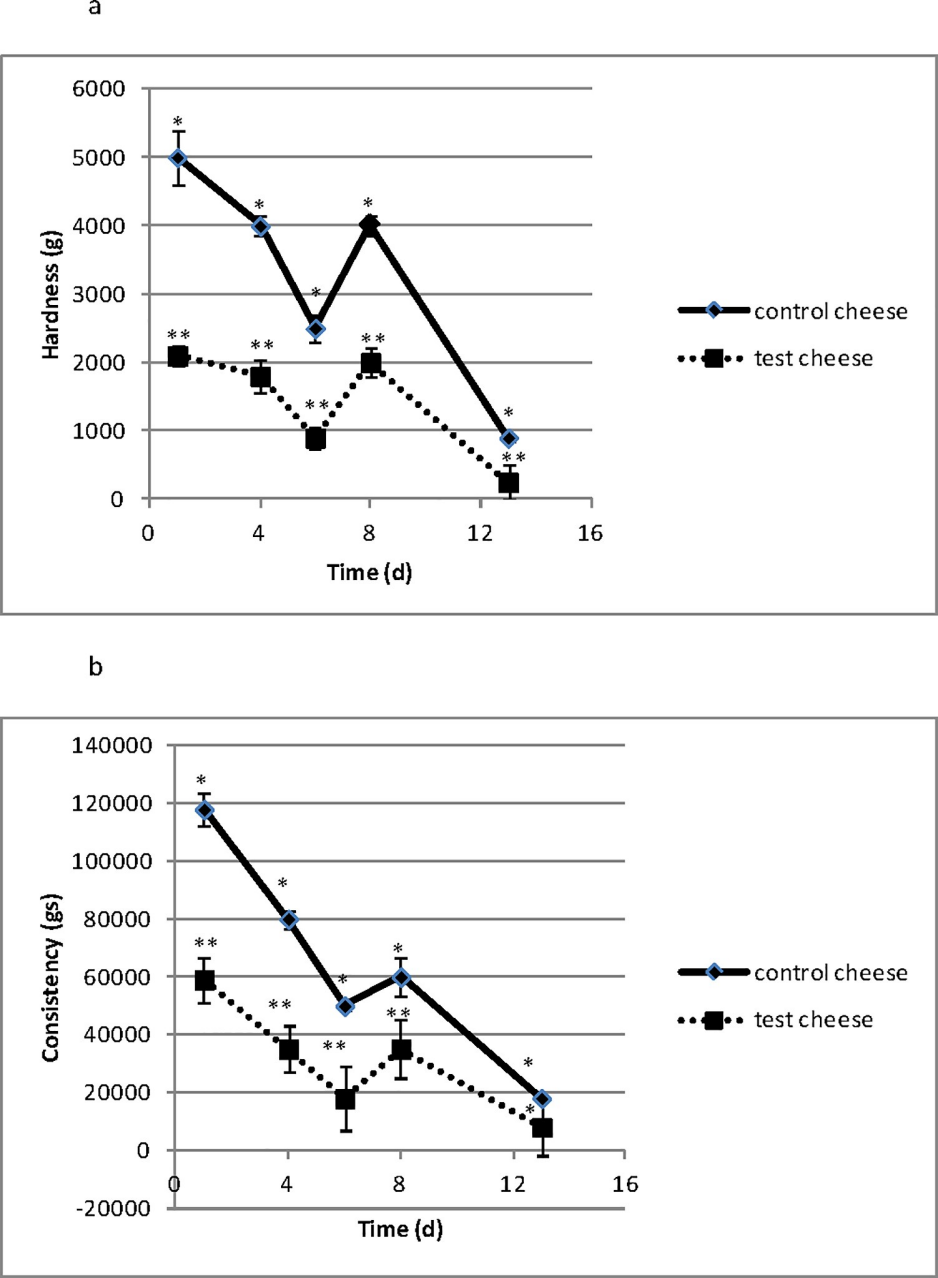
Textural analyses of hardness (a) and consistency (b) evaluated in control and test Squacquerone cheeses during the refrigerated storage (1, 4, 6, 8, 13 days). Results are expressed as mean ± standard deviation. For the statistical analysis, control and test cheeses were compared at each sampling time. Comparing the same day of sampling, textural values of control and test cheeses having different capital letters were significant different (p<0.05).

The textural analyses of hardness and consistency over refrigerated storage is reported in Fig 5, respectively. Regarding hardness, significant differences between the two types of Squacquerone cheese were assessed for any storage time considered (1–13 days). Consistency was found significantly different between the two cheese products up to 8 days of refrigerated storage.

### Evaluation of the fate of test cheese Lactobacilli population (mainly represented by *L*. *crispatus* BC4) in simulated stomach-duodenum passage and in the Simulator of the Human Intestinal Microbial Ecosystem (SHIME)

A simulation of the passage of the test cheese through stomach and duodenum was carried out, and the surviving levels of Lactobacilli (mainly represented by *L*. *crispatus* BC4) were evaluated. The presence of *L*. *crispatus* was verified by genomic DNA extraction from colonies and sequencing the 16S rRNA region according to the method previously indicated.

**Fig 6:**
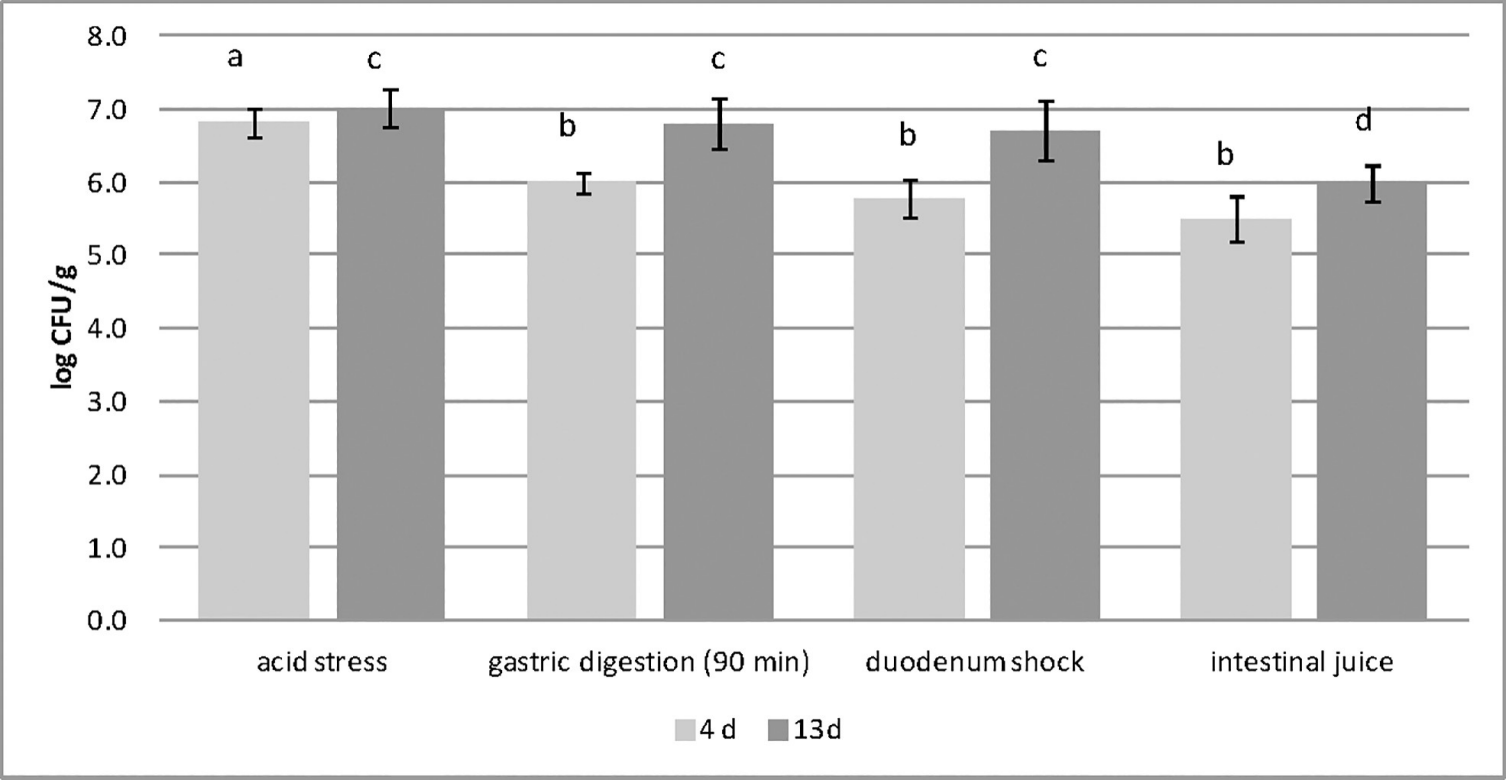
Evolution of *Lactobacillus* population cell loads in test Squacquerone cheese at 4 and 13 days of refrigerated storage, subjected to simulated stomach-duodenum passage. Cell loads are expressed as mean Log CFU/g cheese ± standard deviation. The cell load values recorded after 4 days of cheese refrigeration was statistically compared in relation to the stress applied. Values significant different are highlighted with different superscript letter (p<0.05). The cell load values recorded after 13 days of cheese refrigeration was statistically compared in relation to the stress applied. Values significant different are highlighted with different superscript letter (p<0.05).

At 4 and 13 days of refrigerated storage, test cheese were subjected to different chemical stresses. The simulated physiological stresses applied on the test cheese after 4 days of storage showed that *L*. *crispatus* BC4 (added in the test cheese at level of 7 log CFU/g) was more affected by the application of gastric stress conditions than the duodedum stress. In fact, when gastric condition was applied to the test cheese, containing *Lb*. *crispatus BC4*, after 4 days of storage, its Lactobacilli cell load values decreased of 1 log cycle (Fig 6). When the test cheese after 13 days of storage was subjected to the stomach duodenum passage, the viability of Lactobacilli population were more affected by the application of intestinal juice stress.

**Fig 7:**
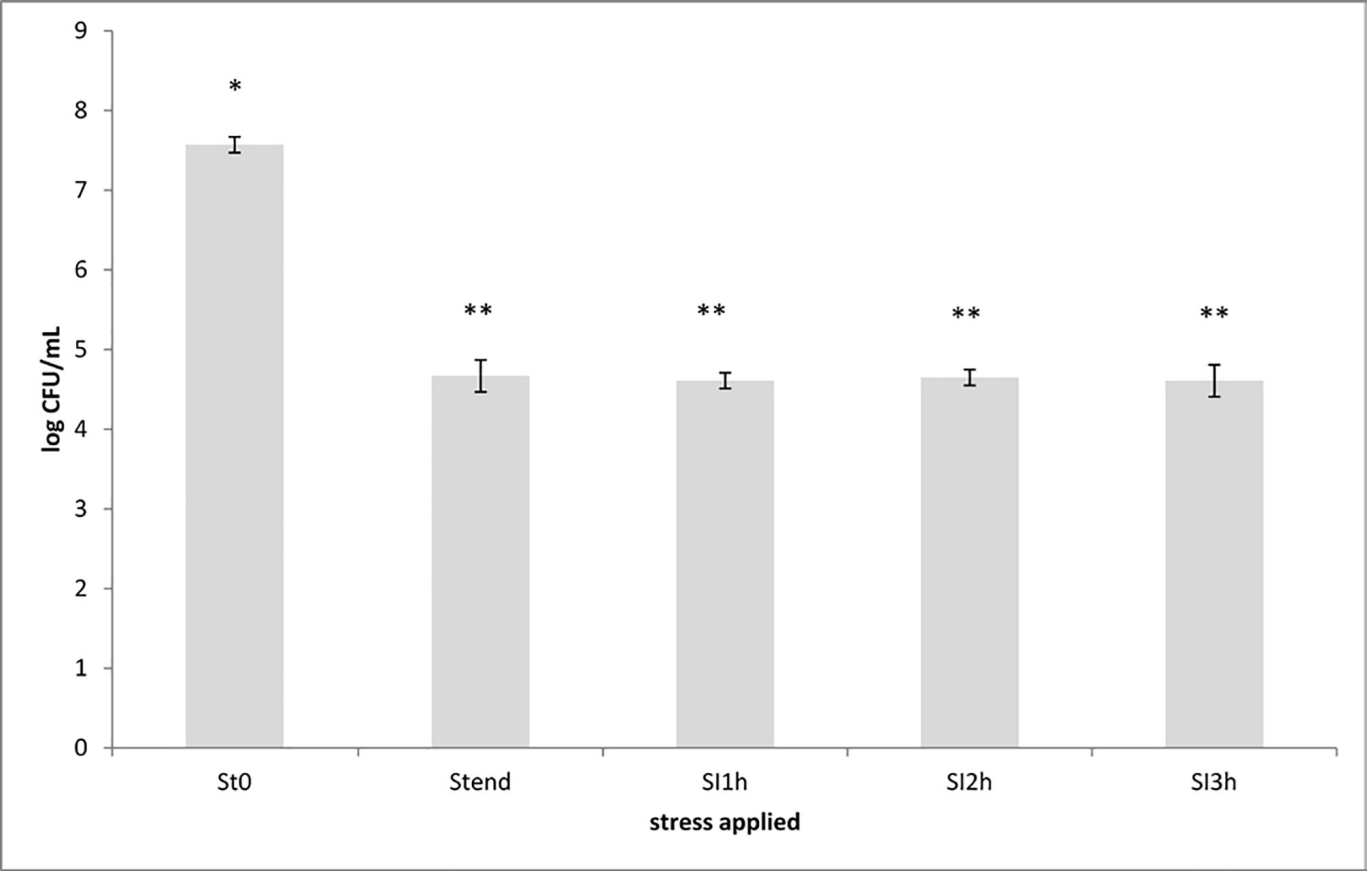
Evolution of *Lactobacillus* population cell loads in test Squacquerone cheese subjected to the Simulator of the Human Intestinal Microbial Ecosystem (SHIME). Plate counts were carried out before (ST0) and after (STend) the stomach condition exposure, and after 1 (SI1), 2 (SI2) and 3 (SI3) h of exposure to the small intestine conditions. Cell loads are expressed as mean Log CFU/mL cheese ± standard deviation. Cell loads values having different symbol are significant different for Lactobacilli population cell loads (p <0.05).

Results related to the survival of the *Lactobacillus* population in the upper gastro intestinal tract under simulated fed conditions in SHIME are shown in Fig 7, which reports the plate count cell loads of lactobacilli detected before and after the exposure to stomach condition, and 1, 2 and 3 hours after the exposure to the small intestine conditions.

Plate count data on MRS medium showed that the transit in the stomach led to a decrease of approx. 3 Log units in lactobacilli viability in the test cheese. During transition in the small intestine, *Lactobacillus* population levels remained constant in the test cheese. The presence of *L*. *crispatus*, at the highest cell loads, was verified by genomic DNA extraction and sequencing the 16S rRNA region according to the method previously indicated. These data showed that the vaginal strain was initially significant affected by the low pH in the stomach while it was resistant to bile salts and pancreatic juices.

## Discussion

The present research is part of a wider project aimed to use food as strategy to increase woman well-being. Generally, since vaginal dysbiosis and other gynaecological infections are generally characterised by a reduction in vaginal lactobacilli content, the administration of probiotics, especially lactobacilli, has been proposed as a valid treatment for vaginal disorders. In fact, a wide literature reports that the administration of probiotics is able to restore the normal vaginal microbiota [53–56]. Beside the vaginal application, a wide literature shows that probiotic bacteria can be administered orally, also through foods, due to their ability to pass from the intestine to the vagina, with a consequent beneficial impact on the vaginal habitat [37–41]. Among probiotic bacteria, *L*. *crispatus*, being routinely found in the vagina of healthy women, is considered one of the most active species in maintaining the eubiosis [30,31,57–59] and several pharmaceutical preparations based on selected strains of *L*. *crispatus* has been proposed and sometimes patented [32,60].

As an alternative to pharmaceutical or nutraceutical preparations, the present research aimed to develop a probiotic soft cheese as dietary strategy to increase woman wellbeing and particularly to prevent or solve dysbiosis disorders.

In this framework the results of the present study show that Squacquerone cheese can represent a suitable carrier of *L*. *crispatus*, able to preserve the strain viability during the refrigerated storage and the simulated gastro intestinal digestion, respecting the hedonistic features of foods. In fact, *L*. *crispatus* BC4 maintained levels of about 7 log CFU/g up to 13 days of refrigerated storage. These cell loads allow a daily intake of 100 g of cheese to introduce the probiotic cell amount recommended by FIL-IDF (International Dairy Federation) that, depending on the probiotic strain, range between 8 and 12 log CFU/day. Consequently, the strain herein considered, fulfills two of the main criteria to select probiotic microorganisms to be included in food systems: i) the compatibility with eventual starter culture during cheese making and ii) the maintenance of cell loads of at least 6 log CFU/g throughout the shelf life [61,62]. In addition, the Squacquerone cheese matrix resulted able to assure the protection of the *L*. *crispatus* against the simulated physiological stresses (low pH, high bile salt concentration, presence of digestive enzymes) characterizing the digestion process. In fact, at the end of the simulated stomach-duodenum passage, the strain showed a viability higher of 6 log CFU/g, independently on the storage time considered. Soft cheeses like Squacquerone or Crescenza are considered optimal carrier and dietary strategy to transfer probiotic strains as they are characterised by a protective protein and fat matrix, neutral or sub-alkaline pH, and high buffering capacity, properties able to protect the probiotic bacteria during their gastro-intestinal transit [47,62,63]. In addition, these cheeses can represent a good source of calcium and vitamins, particularly important for the woman wellbeing. The test cheese containing *L*. *crispatus* BC4 was also subjected to the Simulator of the Human Intestinal Microbial Ecosystem (SHIME). These reactors simulated, over time, firstly the stomach and then the small intestine under fed conditions. Differently from the simulated stomach duodenum passage, the incubation conditions and timing are optimized in the SHIME system in order to resemble *in vivo* situation in the different regions of the gastrointestinal tract. The data obtained confirmed the viability of *L*. *crispatus* BC4, to survive to the *in vivo* stress that the strain can encounter during the digestion process, evidencing its higher sensitiveness toward gastric conditions with respect to bile salts and pancreatic juice.

Notably, the inclusion of the *L*. *crispatus* BC4 improved the organoleptic properties of the cheese in terms of creaminess, flavour and overall acceptance. This is an important feature since the probiotic products are often characterized by lack of good sensory properties and several strategies were proposed to overcome such probiotic food drawbacks [64,65]. In fact, *L*. *crispatus* BC4 strain affected, although at levels compatible with the short ripening time, both the proteolytic and lipolytic patterns of cheese and, consequently, the volatile molecule profiles. These modifications resulted in a product appreciated by the panellists involved in the sensory analysis.

## Conclusions

Although only *in vivo* clinical trials can definitely confirm the functional properties of a food, these data represent the first important tassel in the use of the Squacquerone cheese, containing *L*. *crispatus* BC4, as dietary strategy for woman well-being. The novelty of this work consists in the use of a *Lactobacillus* species, exclusively associated with the vaginal habitat and recognized as healthy marker, for the production of a probiotic food that can promote the woman’s health by preventing gynaecological infections. At present the use of *L*. *crispatus* has been reported only in pharmaceutical formulations; for this reason, our work is a pioneer in the proposal of using this health-promoting vaginal species in the preparation of functional foods of gender.

However, next step of this research will include clinical studies to verify the potential of this functional cheese in humans to prevent vaginal diseases.
